# The Glucose-Lowering Effect of Foxtail Millet in Subjects with Impaired Glucose Tolerance: A Self-Controlled Clinical Trial

**DOI:** 10.3390/nu10101509

**Published:** 2018-10-15

**Authors:** Xin Ren, Ruiyang Yin, Dianzhi Hou, Yong Xue, Min Zhang, Xianmin Diao, Yumei Zhang, Jihong Wu, Jinrong Hu, Xiaosong Hu, Qun Shen

**Affiliations:** 1 Beijing Advanced Innovation Center for Food Nutrition and Human Health, Beijing Technology and Business University, Beijing 100048, China; renxin@btbu.edu.cn (X.R.); zmin@th.btbu.edu.cn (M.Z.); 2 Key Laboratory of Plant Protein and Grain Processing, National Engineering and Technology Research Center for Fruits and Vegetables, College of Food Science and Nutritional Engineering, China Agricultural University, Beijing 100083, China; ruiyang_yin@163.com (R.Y.); xiaozhihou90@126.com (D.H.); xueyong@cau.edu.cn (Y.X.); jihong-wu7268@163.com (J.W.); hujr@cau.edu.cn (J.H.); huxiaos@263.net (X.H.); 3 Chinese Academy of Agricultural Sciences, Beijing 100081, China; xmdiao@yahoo.com.cn; 4 School of Public Health, Peking University, Beijing 100191, China; zhangyumei@bjmu.edu.cn

**Keywords:** dietary intervention, whole foxtail millet, blood glucose, impaired glucose tolerance

## Abstract

Foxtail millet has relatively low starch digestibility and moderate glycemic index compared to other grains. Since there are still no clinical researches regarding its long-term effect on blood glucose, this self-controlled study was conducted to investigate the glucose-lowering effect of foxtail millet in free-living subjects with impaired glucose tolerance (IGT). Fifty g/day of foxtail millet was provided to enrolled subjects throughout 12 weeks and the related clinical parameters were investigated at week 0, 6 and 12, respectively. After 12 weeks of foxtail millet intervention, the mean fasting blood glucose of the subjects decreased from 5.7 ± 0.9 mmol/L to 5.3 ± 0.7 mmol/L (*p* < 0.001) and the mean 2 h-glucose decreased from 10.2 ± 2.6 mmol/L to 9.4 ± 2.3 mmol/L (*p* = 0.003). The intake of foxtail millet caused a significant increase of serum leptin (*p* = 0.012), decrease of insulin resistance (*p* = 0.007), and marginal reduction of inflammation. Furthermore, a sex-dependent difference in glucose-lowering effect of foxtail millet was observed in this study. Foxtail millet could improve the glycemic control in free-living subjects with IGT, suggesting that increasing the consumption of foxtail millet might be beneficial to individuals suffering from type 2 diabetes mellitus.

## 1. Introduction

Diabetes, one of the largest epidemics the world has faced, is a major risk factor for public health. Over the past few decades, both the prevalence of diabetes and the number of diabetics has increased dramatically, due to rapid socioeconomic development and lifestyle changes. A national study from 2007 to 2008 estimated that about 9.7% of the general adult population has been suffering from diabetes, and 15.5% has been suffering from pre-diabetes [1].

Impaired glucose tolerance (IGT) is an intermediate condition in the transition between normality and diabetes. People with IGT are at high risk of progressing to type 2 diabetes (T2DM). The average annual progression rates ranged from 6.0% to 15.7% and the cumulative incidence of diabetes at 6 years reached to 67.7% [2,3]. An unhealthy diet and lack of exercise were associated with a significantly increased risk of diabetes [4,5]. On the contrary, the majority of cases of T2DM could be delayed or prevented by the adoption of a healthy lifestyle, such as sufficient consumption of whole grains, controlled total energy intake, and increased physical activity, resulting in 31–58% of the reduction risk of diabetes [2,3].

Among the established risk factors of T2DM, balanced diet and rational nutrition play an important role [6]. In the past, numerous studies have enhanced our understanding of the relationship between whole grain and glucose metabolism [7]. Prospective studies consistently showed a reduced risk of T2DM with high intakes of whole grains [8]. Fung et al. followed the men from the Health Professionals Follow-up Study without a history of diabetes or cardiovascular disease (*n* = 42898) for ≤12 years and suggested that a diet high in whole grains was associated with a reduced risk of T2DM [9].

Millet is one of the most important drought-resistant whole grain in arid and semiarid areas of Asia and Africa [10]. It has been receiving specific attention because of its excellent nutritive value and potential health benefits such as anti-tumor, anti-oxidant and anti-arteriosclerotic effects [11]. Moreover, millet-based products have markedly slower gastric emptying than rice, potato, or pasta [12]. Previous research has suggested the anti-diabetic effect of finger millet [13] and barnyard millet [14]. However, there are still no clinical researches regarding the glucose-lowering effect of foxtail millet. Our previous study showed that the in vitro starch digestibility of foxtail millet flour was obviously lower than that of wheat flour [15]. Foxtail millet-derived products had a median glycemic index and a gentle stimulation to pancreatic β-cell, which could help diabetics to avoid dangerous spike in blood glucose [15]. These characteristics above-mentioned might contribute to the improvement of postprandial blood glucose in diabetics. In addition, animal experiment also showed that feeding of foxtail millet improved insulin sensitivity and cholesterol metabolism in genetically type 2 diabetic mice [16]. Human clinical trials are necessary to validate the results obtained from in-vitro studies and animal experiments [17].

Based on this, we hypothesized that increasing the intake of foxtail millet could decrease the blood glucose concentrations and improve the blood glucose tolerance. Considering the fact that it is difficult to find a placebo whose color, flavor, texture and taste were almost the same with foxtail millet diet, evaluation of foxtail millet in a randomized clinical trial was not feasible [18]. Taking this into account, we decided to examine the issue in an open-label, self-controlled clinical trial. Thus, 12 weeks of foxtail millet intervention (50 g/day in raw weight) was conducted in free-living subjects with IGT and the effect of foxtail millet diet on glycemic metabolism, lipid metabolism, inflammation status, and body composition was investigated.

## 2. Materials and Methods 

This open-label, self-controlled clinical trial was approved by Ethics Committee of Peking University (IRB00001052-14023). All the subjects were gave written informed consent before participation. The study was performed at the Peking University and registered with Chinese Clinical Trial Registry (No. ChiCTR-OON-16008603). All study performers have completed Good Clinical Practices training and were qualified to conduct clinical research.

### 2.1. Subjects

**Figure 1:**
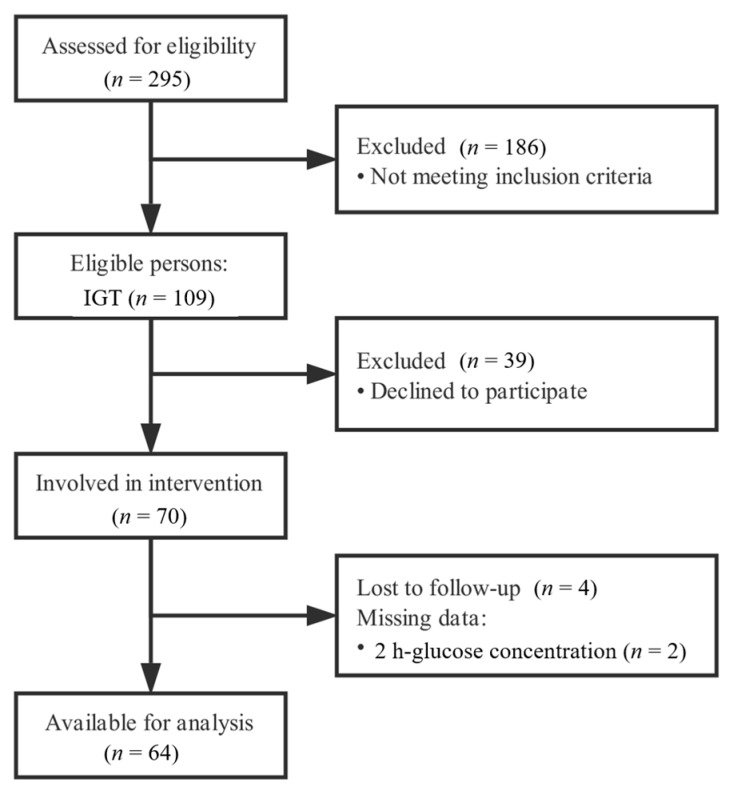
Participant flow diagram.

The 1999 World Health Organization (WHO) diagnostic criteria were used to screen out appropriate subjects based on the criteria of fasting blood glucose (FBG) concentrations <7.0 mmol/L and 2 h-glucose range from 7.8 to 11.1 mmol/L in 75-g oral glucose tolerance test (OGTT) [19]. Eligible subjects were excluded if they were allergic to plant protein or had abnormal renal/liver function or had suffered from a self-reported chronic disease (non-alcoholic fatty liver disease, cancer, immunological disease, etc.) for 6 years and over or were taking medicines known to alter glucose tolerance. Pregnant and breastfeeding women also couldn’t be recruited. It needs to be noted that short-term chronic patients were still included in the present study. After screening 295 participants, a total of 70 eligible subjects participated in the intervention and 64 subjects (27 male subjects and 37 female subjects) were involved in the final analysis (4 subjects failed to follow up and 2 subjects with missing data on 2 h-glucose were excluded, Figure 1). Among the subjects, 69.4% of them were non-smokers and 71.7% of them were non-drinkers. Their average age was 56.0 ± 7.1 years (65.6% of them were over 60 years old), with average body mass index (BMI) of 26.0 ± 3.5 kg/m^2^. Among the female subjects, 67.6% had struggled with gestational diabetes mellitus.

### 2.2. Intervention

**Figure 2:**
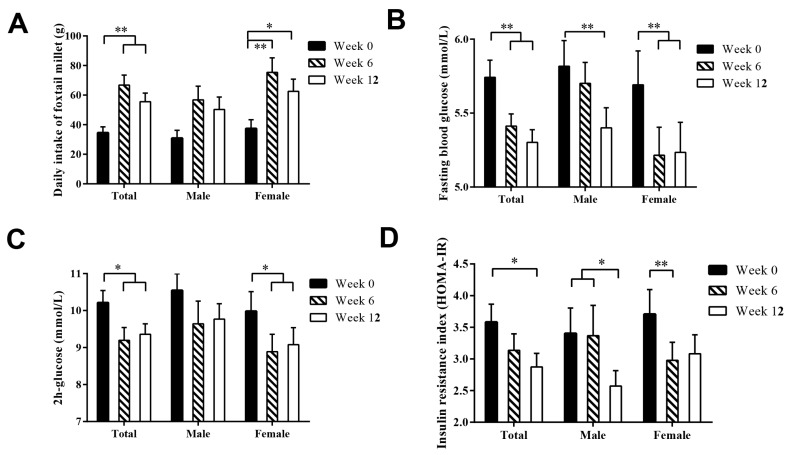
Sex-dependent difference in daily intake of foxtail millet (**A**), fasting blood glucose (**B**), 2 h-glucose (**C**) and insulin resistance index (**D**) in subjects with impaired glucose tolerance. Difference between two different time points was examined using Bonferroni’s post-hoc test. All statistical tests were 2-sided, and * *p* < 0.05 and ** *p* < 0.01 were considered statistically significant.

Foxtail millet steamed bread was cooked and vacuum packaged uniformly, according to the introduction by Ren et al. [20]. A daily intake of 50–150 g of whole grain was recommended by Dietary Guidelines for Chinese Residents (2016). In the present study, 90 g of foxtail millet steamed bread, containing 50 g foxtail millet in raw weight, was provided to participants every day throughout the 12 weeks of intervention. The administered steamed bread was in addition to their habitual daily diet, and the subjects were encouraged to remain on their normal dietary habit aside from substituting equivalent food by steamed bread. To determine compliance, subjects recorded foxtail millet steamed bread consumed each day using a checklist which was returned to the researchers at week 6 and 12, respectively. The amount of foxtail millet intake (Figure 2A) and overall nutrient supply (Table S1) were assessed using 24-h dietary recall at week 0, 6, and 12 respectively [21]. Moreover, the level of physical activity of IGT subjects has been assessed by International Physical Activity Questionnaire (last 7-day recall) according to the standard method [22]. There were no significant differences in the level of physical activity and all kinds of nutrient supply (Table S1). All the subjects backed to Peking University for samples collection at week 0, 6 and 12, respectively. 

### 2.3. Samples Collection and Outcomes Measurement

After overnight fasting, blood samples were collected at 8:00 a.m.–9:30 a.m. at week 0, 6 and 12, respectively. Following centrifugation at 3000× *g* for 15 min, the serum samples were analyzed for fasting blood glucose (FBG), fasting insulin (FINS), fructosamin, fasting C-peptide, total triglyceride (TAG), total cholesterol (TC), high-density lipoprotein cholesterol (HDL-C), low-density lipoprotein cholesterol (LDL-C), apolipoprotein A1 (ApoA1) and apolipoprotein B (ApoB), using an automatic biochemical analyzer (ROCHE COBAS INTEGRA 800, Basel, Switzerland). Several diabetes-related cytokines and hormones, including tumor necrosis factor-α (TNF-α, ERC102a.96, NeoBioscience, Shenzhen, China), interleukin-6 (IL-6, ERC003.96, NeoBioscience), leptin (EZHL-80SK, EMD Millipore, Burlington, MA, USA), adiponectin (EZHADP-61K, EMD Millipore), and glucagon like peptide-1 (GLP-1, SED475Hu, USCN, Wuhan, China), were also detected using the commercially available ELISA kits. In addition, a standard 75-g OGTT [19] was performed and 2 h-blood samples were collected for the detection of 2 h-glucose and 2 h-insulin. Then the insulin resistance index (HOMA-IR, FBG × FINS/22.5) and insulin sensitivity (HOMA-IS, 1/HOMA-IR) were calculated according to Homeostasis Model Assessment respectively [23]. Meanwhile, the blood pressure, body weight, waist circumference and hip circumference were measured using clinical conventional methods. The body composition were examined by bioelectrical impedance analyzer (TBF-410-GS Body Composition Analyzer, Tanita, Tokyo, Japan). All assays and procedures were performed according to the manufacturer’s instructions.

### 2.4. Statistical Analysis

All statistical analyses were performed using SPSS version 20.0 (SPSS Inc., Chicago, IL, USA) and graphs were plotted using GraphPad Prism (Graphpad Software, San Diego, CA, USA). Data were represented as mean ± standard deviation (SD). Significances of treatment effect during intervention period (week 0–week 6–week 12) were accessed by repeated measures analysis of variance (ANOVA). Differences between two different time points were examined using Bonferroni’s post-hoc test Bonferroni’s post-hoc test. All statistical tests were 2-sided, and *p* < 0.05 was considered statistically significant.

## 3. Results

### 3.1. Effect of Foxtail Millet Intervention on Blood Glucose

The intake of foxtail millet induced significant decrease in FBG (*p* < 0.001) and 2 h-glucose (*p* = 0.003) in subjects with IGT (Table 1). After 6 weeks of foxtail millet intervention, the mean FBG decreased by 0.3 ± 0.7 mmol/L (5.7 ± 11.9%) and the mean 2 h-glucose decreased by 1.0 ± 2.7 mmol/L (9.9 ± 25.1%) respectively. Then FBG and 2 h-glucose kept stable until the end of 12 weeks of intervention. However, there were no significant difference of FINS, fructosamine, and 2 h-insulin concentrations during the intervention period (all *p* > 0.05). It was noteworthy that the decreasing tendency of C-peptide was obvious. The HOMA-IR was significantly decreased from 3.6 ± 2.3 at week 0 to 2.9 ± 1.7 at week 12 (decreased by 19.8 ± 46.1%, *p* = 0.015), and the corresponding HOMA-IS increased from 0.4 ± 0.2 at week 0 to 0.5 ± 0.6 at week 12 (increased by 36.7 ± 159.1).

### 3.2. Effect of Foxtail Millet Intake on Blood Lipid and Blood Pressure

According to the baseline data (week 0, Table 1), both the mean blood pressure and mean blood lipid of enrolled subjects had been out of the optimal range and almost reached the high-normal levels, which reminded IGT subjects to pay attention to monitor blood pressure and blood lipid when regulating blood glucose. Although there were no significant changes in TC, TAG, ApoB, and ApoA1/ApoB, the concentrations of HDL-C and ApoA1 were significantly increased at week 6 (both *p* < 0.05) when compared with the baseline. After 12 weeks of foxtail millet intervention, the average of diastolic blood pressure (DBP) was significantly decreased from 84.9 ± 8.5 mmHg to 81.6 ± 7.8 mmHg (*p* = 0.003).

### 3.3. Effect of Foxtail Millet Intake on Anthropometric Indicators

The intake of foxtail millet induced significant decrease of body weight (*p* = 0.004), BMI (*p* < 0.001) and obesity degree (*p* = 0.005). The average of body fat mass decline from 22.1 ± 7.1 kg to 21.1 ± 6.2 kg, which seems in line with the overall change in body weight from initially 69.1 ± 11.6 kg to finally 68.2 ± 11.2 kg. Interestingly, there were no significant changes in body muscle mass, contents of proteins and minerals, waist and hip circumferences after the 12 weeks of intervention (all *p* > 0.05, Table 1).

### 3.4. Effect of Foxtail Millet Intake on Cytokines

The concentrations of serum leptin (Table 1) increased gradually with time of intervention. At the end of the 12 weeks of foxtail millet intervention, the average concentrations of leptin were significantly increased from 8.3 ± 6.4 ng/mL to 9.6 ± 7.0 ng/mL (*p* = 0.012). When compared with the baseline data, the concentrations of IL-6 at week 6 were significantly decreased by 1.5 ± 5.5 pg/mL (*p* = 0.031). Meanwhile, the concentrations of TNF-α at week 6 were also lower than that at week 0 (by 1.4 ± 6.0 pg/mL). However, 12 weeks of foxtail millet intervention did not induce any statistically significant difference in the concentrations of adiponectin and GLP-1.

### 3.5. Sex-Dependent Difference in the Glucose-Lowering Effect of Foxtail Millet

The sex-dependent difference in glucose-lowering effect of foxtail millet was analyzed based on three representative indices of glucose metabolism: FBG, 2 h-glucose and HOMA-IR. There were no significant difference in the above 3 indices between male and female subjects at week 0. During the intervention, the average concentrations of FBG (Figure 2B) in female subjects were significantly decreased at week 6 and then maintained at an almost constant low concentrations throughout 12 weeks. However, the average concentration of FBG in male subjects decreased gradually with time of intervention. The statistically significant change of FBG did not appear until week 12. The change of 2 h-glucose (Figure 2C) was similar to that of FBG both in male and female subjects except that there was no significant decrease in male throughout 12 weeks. As to HOMA-IR (Figure 2D), the significant decrease occurred at first 6 weeks (from week 0 to week 6) in female subjects, while it occurred at last 6 weeks (from week 6 to week 12) in male subjects.

## 4. Discussion

FBG and 2 h-glucose were two of the most crucial criteria for assessing blood glucose homeostasis. The mean FBG and 2 h-glucose of enrolled subjects fully met the diagnosis criteria of IGT (WHO, 1990). Intake of 50 g of foxtail millet per day resulted in significant reduction of blood glucose in subjects with IGT. After 6 weeks of foxtail millet intervention, the mean FBG decreased by 5.7 ± 11.9% and the mean 2 h-glucose decreased by 9.9 ± 25.1%, respectively. Similar glycemic response was also found in aqueous extract of foxtail millet [24] and foxtail millet dosa (a traditional Indian foxtail millet-based breakfast) [25]. Although the rapid decrease did not sustain in the next 6 weeks, both the FBG and 2 h-glucose stayed at almost constant low concentrations with time of intervention. Similar variation trend has also been found in several lifestyle interventions [26]. Poor subject compliance and persistence to a specified diet, which can be proved by the daily intake amount of foxtail millet (Figure 2A), might be one of the main reasons for such trend [27]. The effect of foxtail millet intervention on blood glucose might be explained by the slow digestion of carbohydrate and moderate glycemic index [15]. In addition, the bioactivated fibers, flavonoids, polyphenols and other phytochemicals in foxtail millet might be other contributors to its glucose lowering effect [10,28].

T2DM occurred either when the pancreas could not produce enough insulin (insulin hyposecretion), or when the body cannot effectively use the insulin it produces (insulin resistance). The baseline data from HOMA-IR indicated the insulin resistance was quite common in the enrolled subjects. In accordance with the improvement of HOMA-IR and HOMA-IS, the decrease of fasting C-peptide, one of indicators on the reduction of insulin secretion, suggested that less insulin was needed to regulate blood glucose levels. Therefore, our results showed that there had been a substantial improvement in insulin sensitivity and insulin resistance, resulting from foxtail millet intervention.

Interestingly, Men and women differ in the risk of diabetes mellitus, complications and diabetes management because of their difference in body structure, physiology foundation basis and socioeconomic status [1]. It is necessary to adopt a sex-sensitive approach to optimally prevent and treat diabetes [29]. The response sensitivities of FBG, 2 h-glucose and HOMA-IR of female subjects were much higher than those of male subjects in our present study. Two reasons were presented here for discussion. Firstly, according to the average intake amount of foxtail millet (Figure 2A), men were less compliant on intervention than women [30], which might induce insufficient statistical power. It was difficult to maintain a high compliance rate when assigning participants to a diet that differs from their usual diet [31]. Regardless of the dietary regimen prescribed, the compliance of the participant generally decrease over time [32]. Maintaining subject compliance has been one of challenges when implementing a dietary intervention [33]. In the present study, the average compliance rate was 88.5% (88.9% in men and 88.2% in women, respectively) in the first 6 weeks and 93.0% (91.3% in men and 94.1% in women, respectively) in the last 6 weeks. Specifically, 70.4% participants (62.5% in men and 76.7% in women) insisted on dietary intake of 50 g of foxtail millet in the first 6 weeks, and 58.5% participants (52.4% in men and 62.5% in women) insisted throughout the trial. Secondly, the difference of hormones between men and women also should be considered. It has been reported that hormone replacement therapy improved glycemic control in diabetic postmenopausal women [34] and testosterone replacement therapy improved insulin resistance in diabetic hypogonadal men [35]. Accordingly, foxtail millet intake might promote the secretion of estrogen in women and then improve the glucose control [36]. Of course, more research is needed to confirm this hypothesis. Furthermore, there are many different pathophysiological and social factors involving in the sex-dependent association of millet consumption and glucose metabolism, which could not be specifically determined in our present study.

Besides lowering blood glucose, foxtail millet intake also improved blood pressure and lipid profile. Several researches have suggested that replacement of saturated fatty acid with unsaturated fatty acid led to significant improvement of lipid profile in persons with or without hyperlipidemia [37,38]. Thus, the high amount of unsaturated fatty acid in foxtail millet, especially linolenic acid (C18:2, account for 56.0%) and linoleic acid (C18:1, account for 21.0%), might be the primary contributor to increase of HDL-C and apolipoprotein A1 in IGT subjects. Moreover, our previous study has shown that ingestion of foxtail millet protein hydrolysates ameliorated hypertension in spontaneously hypertensive rats [39]. The significant decrease of DBP in IGT subjects suggested that increasing foxtail millet consumption might be contributing to reducing the risk of hypertension.

It has been proved that being overweight or obese is an independent risk factor for T2DM. Several lifestyle interventions also used body weight loss as a primary outcome measure [3,26]. The significant decrease of body weight, BMI and obesity degree of enrolled subjects in this study indicated that there has been a negative energy balance over the study period. Of course, we could not deny the fact that positive changes observed and attributed to “the glucose lowering effect of foxtail millet” were one of the results of weight loss. Interestingly, contrary to the significant decrease in body fat mass (week 0 vs. week 12, *p* = 0.001), there was no significant difference in body muscle mass, which suggested that foxtail millet may specifically act on the body fat, rather than body protein and minerals. Foxtail millet might be serviced as a kind of healthy food for overweight people.

We also tried to analysis the mechanism of glucose-lowering effect of foxtail millet by investigating cytokines and inflammatory factors. Leptin is an important adipokine, which plays an important role in blood glucose metabolism by suppression of hunger and inhibition of energy intake [40]. The results from our study suggested that increased leptin concentrations, which could reduce appetite, might be a way to improve blood glucose metabolism. Low-grade inflammation is a common feature in subjects with T2DM [41]. The decrease of plasma concentration of IL-6 and TNF-α during intervention might be resulted from bioactivated fibers, flavonoids, polyphenols and other phytochemicals in foxtail millet [28]. Recent data have revealed that the increased concentrations of IL-6 and TNF-α might suppress insulin signal transduction and then interfere with insulin action [42]. Thus, the reducing inflammation, which could enhance insulin action, might be another way to improve blood glucose metabolism. 

According to the published information, this was the first time that the effect of foxtail millet, a kind of whole grain, on glycemic control in subjects with IGT was investigated. Parameters directly (blood glucose, insulin, HOMA-IS, etc.) and indirectly (inflammatory markers, blood pressure, blood lipid, body composition, etc.) related to glycemia were fully investigated at week 0, 6, and 12 respectively. Our positive results might provide an additional dietary option for diabetics and promote the palpability of millet products at both personal and manufacturing levels. However, several limitations should be considered when interpreting the results of this study. Firstly, a main methodological limitation was the self-controlled design, in which part of observed treatment effect may be due to the phenomenon of “regression toward the mean” and also there is some degree of “Hawthorne effect” [43]. Also as mentioned earlier, the methodological difficulties to find a suitable placebo make it unfeasible to conducting a randomized clinical trial. Given this, the self-controlled clinical trail was also used to investigate low-level laser for treatment of tinnitus [43] and aloe vera for prevention of radiation-induced dermatitis [18]. Another limitation of the study was the self-reported amount of foxtail millet consumption, which can increase the level of measurement error and substantially attenuate the strength of the associations [31]. Furthermore, whole foxtail millet was investigated in this study, thus it was difficult to distinguish the specific components of foxtail millet that could account for the glucose-lowering effect. There was strong evidence suggesting that increased consumption of whole grains, as opposed to individually isolated nutrients or supplementations, are associated with reduced risk of developing T2DM [6]. The synergistic actions of bioactive dietary components, including micronutrients and phytochemicals was considered the major contributor to the health benefits of whole grains [7]. Thus, the whole foxtail millet, rather than individual component, was investigated in this study. Additionally, this study was focus on the analysis of physiological outcomes. The possible mechanisms by which foxtail millet improve blood glucose metabolism were not fully investigated. Thereafter, the effects and mechanisms of individual components of foxtail millet on blood glucose metabolism will be further investigated. Considering the fact that HbA1c has strong predictive value for diabetes complications and higher HbA1c indicates worse glycemic control [44]. We will evaluate this variable in the future study as well.

## 5. Conclusions

In 2016, the WHO published the first “Global report on diabetes”, highlighting the serious situation of diabetes around the world and the necessity of developing effective way to reduce the risk of T2DM. The intake of 50 g of foxtail millet per day significantly improved the glycemic control, especially the postprandial glucose, in free-living subjects with IGT. The glucose-lowering effect of foxtail millet might be a result of the interaction of increased leptin concentrations, decreased insulin resistance and reduced inflammation. Although foxtail millet was investigated in this study, it should not be excluded that other whole grain foods also have similar blood glucose-lowering effect. Thus, it is suggested that modern people should appropriately increase and insist on the intake of whole grains.

## Figures and Tables

**Table 1:** T Metabolic characteristics of subjects during study period ^1^.

Indices	Week 0	Week 6	Week 12	*p*-Value ^2^
**Blood glucose**				
FBG (mmol/L)	5.7 ± 0.9 ^a^	5.4 ± 0.7 ^b^	5.3 ± 0.7 ^b^	<0.001
2 h-Glucose (mmol/L)	10.2 ± 2.6 ^a^	9.2 ± 2.8 ^b^	9.4 ± 2.3 ^b^	0.003
FINS (pmol/L)	94.7 ± 49.2	89.3 ± 52.4	84.0 ± 47.5	0.141
2 h-Insulin (pmol/L)	805.3 ± 540.0	736.7 ± 581.1	759.1 ± 460.3	0.529
Fasting C-peptide (pmol/L)	657.4 ± 276.4	626.9 ± 289.0	598.9 ± 256.9	0.149
Fructosamine (μmol/L)	264.6 ± 25.4	268.7 ± 23.4	266.4 ± 26.1	0.293
HOMA-IR	3.6 ± 2.3 ^a^	3.1 ± 2.1 ^ab^	2.9 ± 1.7 ^b^	0.007
HOMA-IS	0.4 ± 0.2	0.4 ± 0.2	0.5 ± 0.6	0.085
Blood lipid				
TC (mmol/L)	5.2 ± 1.0	5.2 ± 1.0	5.2 ± 1.0	0.953
TAG (mmol/L)	2.0 ± 1.2	2.0 ± 1.2	2.1 ± 1.1	0.421
HDL-C (mmol/L)	1.2 ± 0.3 ^a^	1.3 ± 0.3 ^b^	1.3 ± 0.3 ^a^	0.044
LDL-C (mmol/L)	3.3 ± 0.9	3.4 ± 0.9	3.2 ± 0.9	0.056
ApoA1 (mmol/L)	1.6 ± 0.3 ^a^	1.6 ± 0.3 ^b^	1.6 ± 0.3 ^ab^	0.013
ApoB (mmol/L)	1.0 ± 0.3	1.1 ± 0.2	1.0 ± 0.2	0.140
ApoA1/ApoB	1.6 ± 0.6	1.6 ± 0.4	1.6 ± 0.5	0.684
Blood pressure				
SBP (mmHg)	125.0 ± 13.2	126.6 ± 14.5	128.3 ± 13.2	0.155
DBP (mmHg)	84.9 ± 8.5 ^a^	83.9 ± 8.4 ^a^	81.6 ± 7.8 ^b^	0.003
**Cytokine**				
TNF-α (pg/mL)	2.6 ± 5.5	1.3 ± 0.6	1.4 ± 0.5	0.088
IL-6 (pg/mL)	6.2 ± 9.4 ^a^	4.2 ± 5.3 ^b^	4.8 ± 5.5 ^ab^	0.084
**Hormones**				
Leptin (ng/mL)	8.3 ± 6.4 ^a^	9.0 ± 6.1 ^ab^	9.6 ± 7.0 ^b^	0.012
Adiponectin (ug/mL)	2.1 ± 1.6	1.8 ± 1.6	1.8 ± 2.1	0.301
GLP-1 (pg/mL)	23.6 ± 6.1	23.9 ± 7.0	24.8 ± 9.0	0.437
**Anthropometric indices**				
Weight (kg)	69.1 ± 11.6 ^a^	69.4 ± 11.7 ^ab^	68.2 ± 11.2 ^b^	0.004
BMI	26.0 ± 3.5 ^a^	25.8 ± 3.4 ^ab^	25.5 ± 3.2 ^b^	<0.001
Waist circumference (cm)	90.3 ± 8.7	90.4 ± 9.5	89.6 ± 8.5	0.296
Hip circumference (cm)	100.3 ± 7.7	100.0 ± 7.1	100.0 ± 6.9	0.400
Waist-hip ratio	0.9 ± 0.1	0.9 ± 0.1	0.9 ± 0.0	0.644
**Body composition**				
Body fat percentage (%)	31.9 ± 8.1 ^a^	31.4 ± 8.0 ^ab^	30.9 ± 7.7 ^b^	0.132
Body fat mass (kg)	22.1 ± 7.1 ^a^	21.9 ± 7.2 ^ab^	21.1 ± 6.2 ^b^	0.086
Fat-free mass (kg)	47.0 ± 9.4	47.4 ± 9.2	47.2 ± 9.7	0.670
Obesity degree	110.0 ± 15.2 ^a^	110.0 ± 15.4 ^a^	108.3 ± 13.8 ^b^	0.005
Muscle mass (kg)	44.4 ± 9.1	44.8 ± 8.8	44.6 ± 9.3	0.677
Protein (kg)	10.5 ± 3.3	10.5 ± 3.1	10.6 ± 3.2	0.585
Mineral (kg)	2.6 ± 0.4	2.6 ± 0.4	2.6 ± 0.4	0.587

^1^ Data were represented as mean ± SD. FBG, fasting blood glucose; FINS, fasting insulin; TC, total cholesterol; TAG, total triglyceride; HDL-C, high-density lipoprotein cholesterol; LDL-C, low-density lipoprotein cholesterol; ApoA1, apolipoprotein A1; ApoB, apolipoprotein B; SBP, systolic blood pressure; DBP, diastolic blood pressure; TNF-α, tumor necrosis factor-α; GLP-1, glucagon-like peptide-1; IL-6, interleukin-6; BMI, body mass index; SD, standard deviation; ANOVA, analysis of variance. ^2^ Significances of treatment effect were accessed by repeated measures ANOVA during the intervention period (week 0–week 6–week 12). ^a,b^ Data with the different superscript letters in the same row differ significantly (*p* < 0.05); Differences between two subgroups were examined using Bonferroni’s post-hoc test.

## Supplementary Materials

The following are available online at http://www.mdpi.com/2072-6643/10/10/1509/s1, Table S1: The overall nutrient supply and level of physical activity of subjects.

## References

[1] Yang W., Lu J., Weng J., Jia W., Ji L., Xiao J., Shan Z., Liu J., Tian H., Ji Q. (2010). Prevalence of diabetes among men and women in China. N. Engl. J. Med..

[2] Pan X.-R., Li G.-W., Hu Y.-H., Wang J.-X., Yang W.-Y., An Z.-X., Hu Z.-X., Xiao J.-Z., Cao H.-B., Liu P.-A. (1997). Effects of diet and exercise in preventing NIDDM in people with impaired glucose tolerance: The Da Qing IGT and Diabetes Study. Diabetes Care.

[3] Lindström J., Ilanne-Parikka P., Peltonen M., Aunola S., Eriksson J.G., Hemiö K., Hämäläinen H., Härkönen P., Keinänen-Kiukaanniemi S., Laakso M. (2006). Sustained reduction in the incidence of type 2 diabetes by lifestyle intervention: Follow-up of the Finnish Diabetes Prevention Study. Lancet.

[4] Hu F.B., Manson J.E., Stampfer M.J., Colditz G., Liu S., Solomon C.G., Willett W.C. (2001). Diet, lifestyle, and the risk of type 2 diabetes mellitus in women. N. Engl. J. Med..

[5] Ajala O., English P., Pinkney J. (2013). Systematic review and meta-analysis of different dietary approaches to the management of type 2 diabetes. Am. J. Clin. Nutr..

[6] Ley S.H., Hamdy O., Mohan V., Hu F.B. (2014). Prevention and management of type 2 diabetes: Dietary components and nutritional strategies. Lancet.

[7] Xi P., Liu R.H. (2016). Whole food approach for type 2 diabetes prevention. Mol. Nutr. Food Res..

[8] Cho S.S., Qi L., Fahey G.C., Klurfeld D.M. (2013). Consumption of cereal fiber, mixtures of whole grains and bran, and whole grains and risk reduction in type 2 diabetes, obesity, and cardiovascular disease. Am. J. Clin. Nutr..

[9] Fung T.T., Hu F.B., Pereira M.A., Liu S., Stampfer M.J., Colditz G.A., Willett W.C. (2002). Whole-grain intake and the risk of type 2 diabetes: A prospective study in men. Am. J. Clin. Nutr..

[10] Saleh A.S., Zhang Q., Chen J., Shen Q. (2013). Millet grains: Nutritional quality, processing, and potential health benefits. Compr. Rev. Food Sci. F.

[11] Muninarayana C., Balachandra G., Hiremath S., Iyengar K., Anil N. (2010). Prevalence and awareness regarding diabetes mellitus in rural Tamaka, Kolar. Int. J. Diabetes Dev. Ctries.

[12] Cisse F., Erickson D.P., Hayes A., Opekun A.R., Nichols B.L., Hamaker B.R. (2018). Traditional Malian Solid Foods Made from Sorghum and Millet Have Markedly Slower Gastric Emptying than Rice, Potato, or Pasta. Nutrients.

[13] Kumari P.L., Sumathi S. (2002). Effect of consumption of finger millet on hyperglycemia in non-insulin dependent diabetes mellitus (NIDDM) subjects. Plant Food Hum. Nutr..

[14] Ugare R., Chimmad B., Naik R., Bharati P., Itagi S. (2014). Glycemic index and significance of barnyard millet (Echinochloa frumentacae) in type II diabetics. J. Food Sci. Technol..

[15] Ren X., Chen J., Molla M.M., Wang C., Diao X., Shen Q. (2016). In vitro starch digestibility and in vivo glycemic response of foxtail millet and its products. Food Funct..

[16] Choi Y.-Y., Osada K., Ito Y., Nagasawa T., Choi M.-R., Nishizawa N. (2005). Effects of dietary protein of Korean foxtail millet on plasma adiponectin, HDL-cholesterol, and insulin levels in genetically type 2 diabetic mice. Biosci. Biotechnol. Biochem..

[17] Faggion C.M., Schmitter M., Tu Y.-K. (2009). Assessment of replication of research evidence from animals to humans in studies on peri-implantitis therapy. J. Dent..

[18] Haddad P., Amouzgar-Hashemi F., Samsami S., Chinichian S., Oghabian M.A. (2013). Aloe vera for prevention of radiation-induced dermatitis: A self-controlled clinical trial. Curr. Oncol..

[19] World Health Organization Definition, Diagnosis and Classification of Diabetes Mellitus and Its Complications. Part 1: Diagnosis and Classification of Diabetes Mellitus. http://apps.who.int/iris/bitstream/handle/10665/66040/WHO_NCD_NCS_99.2.pdf;jsessionid=9C91D94A2CCE91A06F9E772A20ABC984?sequence=1.

[20] Ren X., Chen J., Wang C., Molla M.M., Diao X., Shen Q. (2016). In vitro starch digestibility, degree of gelatinization and estimated glycemic index of foxtail millet-derived products: Effect of freezing and frozen storage. J. Cereal Sci..

[21] Xue Y., Lee E., Ning K., Zheng Y., Ma D., Gao H., Yang B., Bai Y., Wang P., Zhang Y. (2015). Prevalence of picky eating behaviour in Chinese school-age children and associations with anthropometric parameters and intelligence quotient. A cross-sectional study. Appetite.

[22] Fan M., Lyu J., He P. (2014). Chinese guidelines for data processing and analysis concerning the International Physical Activity Questionnaire. Zhonghua Liu Xing Bing Xue Za Zhi.

[23] Matthews D.R., Hosker J.P., Rudenski A.S., Naylor B.A., Treacher D.F., Turner R.C. (1985). Homeostasis model assessment: Insulin resistance and β-cell function from fasting plasma glucose and insulin concentrations in man. Diabetologia.

[24] Sireesha Y., Kasetti R.B., Nabi S.A., Swapna S., Apparao C. (2011). Antihyperglycemic and hypolipidemic activities of Setaria italica seeds in STZ diabetic rats. Pathophysiology.

[25] Narayanan J., Sanjeevi V., Rohini U., Trueman P., Viswanathan V. (2016). Postprandial glycaemic response of foxtail millet dosa in comparison to a rice dosa in patients with type 2 diabetes. Indian J. Med. Res..

[26] Lindström J., Louheranta A., Mannelin M., Rastas M., Salminen V., Eriksson J., Uusitupa M., Tuomilehto J. (2003). The Finnish Diabetes Prevention Study (DPS) Lifestyle intervention and 3-year results on diet and physical activity. Diabetes Care.

[27] Nutrition D., Trial C. (2006). adherence to the ADA nutritional recommendations, targets of metabolic control, and onset of diabetes complications. A 7-year, prospective, population-based, observational multicenter study. J. Diabetes Complicat..

[28] Sharma N., Niranjan K. (2017). Foxtail millet: Properties, processing, health benefits and uses. Food Rev. Int..

[29] Harreiter J., Kautzkywiller A. (2018). Sex and Gender Differences in Prevention of Type 2 Diabetes. Front. Endocrinol..

[30] Johansson E., Long N., Diwan V., Winkvist A. (1999). Attitudes to compliance with tuberculosis treatment among women and men in Vietnam. Int. J. Tuberc. Lung Dis..

[31] Pan A., Lin X., Hemler E., Hu F.B. (2018). Diet and Cardiovascular Disease: Advances and Challenges in Population-Based Studies. Cell. Metab..

[32] Coles L.T., Fletcher E.A., Galbraith C.E., Clifton P.M. (2014). Patient freedom to choose a weight loss diet in the treatment of overweight and obesity: A randomized dietary intervention in type 2 diabetes and pre-diabetes. Int. J. Behav. Nutr. Phys. Act..

[33] Crichton G.E., Howe P.R.C., Buckley J.D., Coates A.M., Murphy K.J., Bryan J. (2012). Long-term dietary intervention trials: Critical issues and challenges. Trials.

[34] Crespo C.J., Smit E., Snelling A., Sempos C.T., Andersen R.E. (2002). Hormone replacement therapy and its relationship to lipid and glucose metabolism in diabetic and nondiabetic postmenopausal women results from the third national health and nutrition examination survey (NHANES III). Diabetes Care.

[35] Kapoor D., Goodwin E., Channer K., Jones T. (2006). Testosterone replacement therapy improves insulin resistance, glycaemic control, visceral adiposity and hypercholesterolaemia in hypogonadal men with type 2 diabetes. Eur. J. Endocrinol..

[36] Franconi F., Campesi I., Occhioni S., Tonolo G. (2012). Sex-gender differences in diabetes vascular complications and treatment. Endocr. Metab. Immune Disord. Drug Targets.

[37] Sirtori C.R., Crepaldi G., Manzato E., Mancini M., Rivellese A., Paoletti R., Pazzucconi F., Pamparana F., Stragliotto E. (1998). One-year treatment with ethyl esters of *n*-3 fatty acids in patients with hypertriglyceridemia and glucose intolerance: reduced triglyceridemia, total cholesterol and increased HDL-C without glycemic alterations. Atherosclerosis.

[38] Gardner C.D., Kraemer H.C. (1995). Monounsaturated versus polyunsaturated dietary fat and serum lipids. A meta-analysis. Arterioscler. Thromb. Vasc. Biol..

[39] Jing C., Wei D., Xin R., Chao W., Pan Z., Diao X., Shen Q. (2016). Effect of foxtail millet protein hydrolysates on lowering blood pressure in spontaneously hypertensive rats. Eur. J. Nutr..

[40] Jéquier E. (2002). Leptin signaling, adiposity, and energy balance. Ann. N. Y. Acad. Sci..

[41] Call M.C. (2012). Inflammation and type 2 diabetes. Diabetes Metab..

[42] Dandona P., Aljada A., Bandyopadhyay A. (2004). Inflammation: The link between insulin resistance, obesity and diabetes. Trends Immunol..

[43] Okhovat A., Berjis N., Okhovat H., Malekpour A., Abtahi H. (2011). Low-level laser for treatment of tinnitus: A self-controlled clinical trial. J. Res. Med. Sci..

[44] Stratton I.M., Adler A.I., Neil H.A.W., Matthews D.R., Manley S.E., Cull C.A., Hadden D., Turner R.C., Holman R.R. (2000). Association of glycaemia with macrovascular and microvascular complications of type 2 diabetes (UKPDS 35): Prospective observational study. BMJ.

