# Supplementary material for: The Glucose-Lowering Effect of Foxtail Millet in Subjects with Impaired Glucose Tolerance: A Self-Controlled Clinical Trial
Source: Nutrients. 2018 Oct 15;10(10):1509. doi: 10.3390/nu10101509 (PMC6213109; doi:10.3390/nu10101509)
Supplement: Supplementary file 1 [file nutrients-10-01509-s001.pdf]

Table S1. The overall nutrient supply and level of physical activity of subjects

Indices	Week 0	Week 6	Week 12	P-value [1]
**Nutrient supply**				
Energy (KJ/day)	6,868.9 ± 2,834.62	7,370.7 ± 2,840.3	7,162.7 ± 2,784.6	0.585
Protein (g/day)	58.6 ± 27.60	62.5 ± 28.4	59.8 ± 28.4	0.67
Fat (g/day)	60.9 ± 32.94	65.8 ± 36.0	57.4 ± 26.3	0.418
Carbohydrate (g/day)	216.5 ± 102.70	241.1 ± 94.1	242.6 ± 103.1	0.118
Cholesterol (mg/day)	11.4 ± 6.61	13.2 ± 7.9	13.8 ± 10.3	0.213
Ash (g/day)	357.4 ± 342.85	327.8 ± 266.7	291.6 ± 288.0	0.438
Vitamin A (mg/day)	21.2 ± 34.84	18.6 ± 8.1	20.7 ± 15.5	0.721
Tot carotene (mg/day)	614.6 ± 1,288.88	555.3 ± 571.7	411.6 ± 292.4	0.338
Thiamine (mg/day)	1,574.5 ± 1,810.94	2,112.0 ± 2,817.5	1,680.0 ± 1,611.9	0.708
Riboflavin (mg/day)	0.8 ± 0.59	1.0 ± 0.5	1.0 ± 0.9	0.219
Niacin (mg/day)	1.0 ± 0.67	1.2 ± 0.6	0.9 ± 0.5	0.102
Vitamin C (mg/day)	12.4 ± 8.63	12.7 ± 7.6	14.6 ± 12.9	0.396
Vitamin E (mg/day)	78.4 ± 84.43	103.2 ± 78.3	82.7 ± 53.9	0.351
Ca (mg/day)	30.9 ± 16.17	32.9 ± 22.6	33.6 ± 18.3	0.673
P (mg/day)	498.2 ± 328.89	599.1 ± 353.1	585.7 ± 339.2	0.077
K (mg/day)	945.6 ± 578.10	1,029.4 ± 490.5	1,019.5 ± 509.7	0.609
Na (mg/day)	1,685.3 ± 1,072.25	2,050.9 ± 1,023.3	2,104.6 ± 1,738.6	0.201
Mg (mg/day)	3,764.2 ± 1,727.54	3,936.8 ± 2,257.2	3,729.3 ± 1,616.4	0.388
Fe (mg/day)	305.6 ± 343.92	309.8 ± 136.2	333.9 ± 226.2	0.699
Zn (mg/day)	21.8 ± 22.13	23.9 ± 14.5	24.0 ± 17.2	0.748
Se (mg/day)	9.0 ± 4.76	9.9 ± 4.8	10.0 ± 5.0	0.288
Cu (mg/day)	41.3 ± 20.98	44.8 ± 23.8	42.8 ± 23.3	0.451
Mn (mg/day)	2.0 ± 1.83	1.9 ± 1.1	2.2 ± 1.6	0.345
Retinol (μg/day)	4.3 ± 3.15	4.4 ± 2.2	5.1 ± 3.0	0.213
**Physical activity**				
level of physical activity (MET min/w)	2,287 ± 261	2302 ± 293	2456 ± 255	0.394

[1] Significances of treatment effect were accessed by repeated measures ANOVA during the intervention period (week 0–week 6-week 12).

[a, b, c] Data with the different superscript letters in the same row differ significantly ($P < 0.05$); Differences between two subgroups were examined using Bonferroni's post-hoc test.
